# Are there morally relevant differences between hymen restoration and bloodless treatment for Jehovah’s Witnesses?

**DOI:** 10.1186/1472-6939-15-89

**Published:** 2014-12-23

**Authors:** Niklas Juth, Niels Lynøe

**Affiliations:** Stockholm Centre for Healthcare Ethics, Karolinska Institutet, LIME, 171 77 Stockholm, Sweden

**Keywords:** Applied and Professional Ethics, Obstetrics and Gynaecology, Honour, Hymen reconstruction, Virginity

## Abstract

**Background:**

Hymen reconstruction is a controversial measure performed to help young females under threat of honour-related violence. Official guidelines often reject offering hymen reconstructions. On the other hand, extraordinary measures in order to enable operations of Jehovah’s Witnesses who want a bloodless operation in order to avoid religiously related sanctions are often considered praiseworthy. The aim is thus to examine whether or not there are relevant differences between these two measures.

**Discussion:**

We identified twelve potential differences. One difference could be considered relevant (patient-safety), but in favour of hymenoplastic operations.

**Summary:**

Since we did not identify enough relevant differences to justify offering bloodless operations to Jehovah’s Witnesses but not offering hymen reconstruction due to honour-related norms, we conclude that these two groups of patients should be treated equally. This means that neither of the patient groups should be offered these extraordinary operations or that both groups of patients should be offered such operations. Similarly, there are no reasons for judging those who perform the operations differently.

## Background

Hymen reconstruction is a controversial measure that is sometimes performed to help young females under threat of honour-related violence. Official guidelines often reject offering hymen reconstructions. On the other hand, extraordinary measures in order to enable operations on Jehovah’s Witnesses who want a bloodless operation in order to avoid religiously related sanctions are often considered praiseworthy. The aim of this article is to examine whether or not there indeed are morally relevant differences between these two measures. These may seem to be two very different practices, but we believe that a comparison between them reveals a number of tacit values in health care that are morally problematic. Hence, we argue that there are morally no relevant differences or, at least, that they are not important enough to justify assessing the two practices as differently as is currently the case.

### Honour-related norms

There are many ways of characterising honour cultures [1]. However, we are above all interested in one feature, namely the primacy of the collective: when one acts contrary to the norms of the culture, one is thought to be disgracing not only oneself, but also the collective, primarily the family, of which one is a part. We are also interested in one honour-related norm, namely the norm of a woman remaining a virgin before marriage: in order to keep agreements about future arranged marriages between families, it is usually presupposed that the young female especially will be unquestionably a virgin on the day of her marriage. Since traditional honour cultures are commonly patriarchal, focus has been on the young females rather than the males. When arranging marriages between families, the whole arrangement might be jeopardised if the heads of the families, i.e. the patriarchs, or other male members, suspect the young female of having been sexually active before marriage. Disgrace is then considered to be brought on the young woman’s family; the family’s honour is questioned. The only way of restoring that honour is commonly thought to be to impose sanctions on the daughter. She can be expelled from home or, at worst, killed. If the patriarch or other male members of the family are unable to defend or restore the family’s honour, the result is *shame*. Shame is acknowledged as one of the most devastating of all emotions [2].

In ‘western’ societies, by contrast, honour-related norms are generally considered obsolete and dysfunctional. When families from traditional honour cultures move to ‘western’ societies, conflicts can arise between family members who differ in their adaptation to the new culture. Some young women adopt the attitudes or behaviour of the host country, including tolerance towards (or engaging in) sexual relations before marriage. Since very few of these young females (who have engaged in premarital sex) are prepared to leave their families, they might turn to a gynaecologist in order to have a so-called hymen reconstruction or restoration by adding a superficial suture [3]. A hymen restoration is supposed to make the young female ‘tighter’ and able to produce a bleeding during the supposedly first intercourse on the wedding night. Although a hymen reconstruction might not always work as expected, the young female may feel confident and safe as a result of the operation [4].

In Sweden at least, a hymen restoration is not considered a routine operation that gynaecologists are supposed to offer females on request. The official guidelines - as issued e.g. by the Governmental body National Centre for Knowledge on Men’s Violence Against Women - say that it has no medical function and is a morally unacceptable operation that preserves a repressive patriarchal tradition [5, 6]. Instead, the female should be informed about her human rights (e.g. the right to have consensual sexual intercourse without being threatened, exposed to violence or killed as a result) and if the young female’s life is threatened, healthcare staffs are supposed to turn to the social authorities and the police [5, 6]. This view is also imposed on the gynaecologist, although sometimes the head of a gynaecological clinic lets the gynaecologist perform hymen reconstruction as long as the gynaecologist keeps a low profile and nothing is made public, even though the gynaecologist considers the operation as being performed on a vital indication, i.e. out of concern for the patient’s life [3, 7].

To sum up, the superficial hymenoplastic of the kind described is considered an *extraordinary* intervention in the merely descriptive sense of not being a part of routine healthcare of the sort any patient with an appropriate need could be expected to be offered (unlike, for instance, antibiotics in the case of pneumonia). Furthermore, at least in some countries (e.g. Sweden), it is officially considered a *questionable* or even *wrongful* intervention, albeit not legally prohibited. In order to see whether this moral evaluation of the intervention is defensible, it is interesting to compare it with another extraordinary intervention where the evaluation is the opposite and investigate whether there are any morally relevant differences between them. In order to understand the intervention and its rationale, we must turn to another sub-culture, namely Jehovah’s Witnesses.

### The norms of Jehovah’s Witnesses

Jehovah’s Witnesses is a Christian religious movement established in the United States during the 1870s [8]. They support the idea of the sanctity of life, which, as they see it, means for instance not participating in war and not accepting abortion. More unusually, blood is considered as life and is as such also included in the idea of the sanctity of life. Jehovah’s Witnesses take this to mean that blood which has left a body should not be transferred to others, neither via the mouth nor via blood-vessels [8]. Accordingly, blood transfusions are prohibited, and those who have accepted a blood transfusion in order to perform a life-saving operation could be expected to be expelled from the Jehovah’s Witnesses. Being expelled from the religious congregation – referred to as disfellowshipment by Jehovah’s Witnesses – might be very troublesome for the disfellowshipped member, since he or she is shunned by the other members in order to keep the congregation free of immoral influence. Shunning makes the excommunicated person isolated, since he or she loses all old friends and relatives. The disfellowshipped person is also supposed to feel shame. It is, at least to some extent, understandable that such a person may prefer to abstain from a life-saving operation rather than be alive under such conditions.

Jehovah’s Witnesses’ special view on blood-transfusions is fairly well-known and acknowledged, at least among healthcare staffs [8]. The case of Jehovah’s Witnesses has actually been a paradigm case for a patient’s right to abstain from medical treatments, including life-saving treatments. The negative right (i.e. the right that corresponds to the duty of others to abstain from acting in certain ways towards the right-holder) to abstain from treatment is today considered to a fairly uncontroversial implication of the right to respect for autonomy [9]. The only situations where the society goes against a Jehovah’s Witnesses’ wish to avoid a life-saving blood transfusion is when it concerns children who have not come of age. In such cases the social authorities are called in and the children are taken care of.

As regards respecting Jehovah’s Witnesses’ *right to refrain from* blood transfusion, there is an obvious difference compared with hymen reconstructions of young females whose lives are threatened by family members due to honour-related norms: such a patient is not asking healthcare personnel to respect her negative rights to abstain from treatment – she is asking for a certain treatment to be performed. However, the cases become more alike when a member of Jehovah’s Witnesses is suffering from a disease for which a non-emergency operation is needed and accordingly where blood transfusion could be avoided if certain preoperative, operative and post-operative treatments are offered [10–12]. Examples include preoperative treatment with erythropoietin combined with iron therapy in order to stimulate the patient’s own production of red blood-cells. Another possible preoperative procedure is haemo-dilution and storage of blood in order to give it back when needed. During the operation the surgeon could also apply blood salvage in order to recycle the cleaned blood. During an operation and afterwards, surgeons could provide blood substitutes (carrying oxygen or otherwise) in order e.g. to prevent shock. A patient suffering from low blood concentration after an operation or just after having been delivered of a child is taken care of in hospital for perhaps several weeks, which is significantly longer than for patients accepting blood transfusions.

### Comparisons of hymen restoration and bloodless operations

As we have seen, healthcare providers are prepared to offer a Jehovah’s Witness quite a lot of special interventions in order to save the patient’s life, interventions that are not routinely offered to patients in healthcare. That is to say, healthcare providers are actually prepared to offer extraordinary medical interventions in order not to upset Jehovah’s Witnesses’ religious norms and beliefs. Why are they not prepared to do the same for females who ask for hymen reconstruction due to honour-related norms and beliefs?

In order to examine this issue we have to focus on potential differences and ascertain whether there are differences and, if so, relevant ones. If we are able to show that there are no relevant differences between the two cases, it seems unavoidable to conclude that they should be treated equally, according to the formal principle of justice that equal cases ought to be treated equally [13]. So if there are no relevant differences and we accept that Jehovah’s Witnesses should be offered extraordinary measures, then we should also offer young females from an honour culture extraordinary measures. Or, alternatively, if we are not prepared to offer the young females extraordinary measures, nor should we offer Jehovah’s Witnesses extraordinary measures. Only if we are able to identify relevant differences in the two cases can we justify treating the two cases differently.

## Discussion

### Potentially relevant differences

In the following, we present some regards in which offering hymen restoration to patients subjected to honour-related threats, on the one hand, and operations without transfusions to patients who are Jehovah’s Witnesses and threatened with shunning, on the other, could be thought to differ. We ask whether they are different indeed and, if so, whether the difference is relevant, i.e., makes it reasonable to treat these two cases differently.

Issue 1. *Is there a need in both cases?* If a sexually active young female is unable to imitate (alleged) signs of virginity and produce a bleeding during the wedding night, her life might be threatened or, perhaps, she may be subject to harassment, shunning and shame. An extraordinary hymenoplastic operation might solve the problem. A Jehovah’s Witness’s life might also be threatened if he/she refuses blood-transfusion or, perhaps, she may have a prolonged period of recovery. An extraordinary bloodless operation might solve the problem. In both cases we are dealing with possibly life-threatening situations, and in both cases the effects of an extraordinary intervention might solve the problem. In this sense there is a need for an operation in both cases. It may be held that there can only be a need for a health care intervention if there are no other (good enough) alternatives. But, as we will argue later, the interventions are similar in this regard as well (Issues 7 and 8). In principle, then, there is no relevant difference in the two cases regarding needs.

Issue 2. *Is there a medical reason in both cases?* It could be argued that there are *medical* reasons for operating on Jehovah’s Witnesses patients, but that the young females from honour cultures have no medical problems in the first place.

However, this line of reasoning conflates different issues. The question here is not whether Jehovah’s Witness patients should be offered an operation for their medical conditions in the first place. Of course they should. The question is whether we should offer *extraordinary* medical measures in order for the patient to be able to live according to their (unusual) norms, measures we do not offer normally. In this regard, both cases are similar.

It could, of course, be argued that healthcare professionals should only treat threats to life that have medical causes and that other authorities should deal with other possible causes of death. Remember that the Jehovah’s Witness patients’ threat to life is due to blood loss from an operation, and the female honour-culture patients’ threat to life hinges on violence from relatives. It is only appropriate that healthcare deal with the former and for other authorities, e.g. the police, deal with the latter.

In remembering this difference it is, however, important not to forget the similarities in what is at stake for the two patient groups. By going to the police, the young female must break with her family and community. Healthcare might be the only institution that can make it possible for the young female to minimise the risk to her life, while at the same time remaining a part of her community. This is equally true of Jehovah’s Witness patients – they can also circumvent the medical need by choosing to leave their community: the need is a result of their resistance to breaking with their community. So if healthcare helps the latter for this reason, why should it not help the former?

Issue 3. *Do the extraordinary interventions differ with respect to patient safety?* Hymen restoration is an easily performed operation and is fairly risk-free [4]. Bloodless operations and the subsequent intensive care might be complicated and risky. Patient safety could be considered a relevant aspect. However, it argues in favour of the superficial hymenoplastic operation rather than the bloodless operation.

Issue 4: *Do the benefits for surgical practice and development differ?* Initially, bloodless surgery was developed as a special strategy in order to avoid blood transfusions for Jehovah’s Witnesses. However, these strategies have also proved useful for other medical purposes, for instance to minimise risks of transmitting infections, e.g., HIV and hepatitis. In this manner, Jehovah’s Witnesses’ norms have spurred a valuable development for surgery in general [14]. Additionally, surgeons have gained the possibility of publishing papers about successful cases [10–12].

In comparison, hymen restorations have not furthered the development of gynaecology. However, it is a widely acknowledged principle of medical ethics that healthcare should not decide which medical procedures to offer on the basis of their potential for developing into useful research for the future, but rather according to what is in the best interests of the individual patient [15]. So this difference does not seem to justify treating them differently in healthcare.

Issue 5. *Are the hymen reconstructions, compared to bloodless operations, merely an expression of so-called medicalization of social problems?* The problem in both cases emanates from norms (honour-related and religious ones), and a medical solution is chosen in both cases. This means that if we would characterise one of the interventions as medicalization of social problems, we should do so in the other case as well. If we accept the premise that in both cases we are dealing with potentially life-threatening situations, the interventions are not futile – there is a need (see issue 1). In this respect there is no (relevant) difference. Moreover, even if there were a difference in this regard, it is far from clear that it is morally relevant, since it is questionable to what extent and in what ways the medicalization of social and other non-medical problems is morally problematic to start with [16].

Issue 6. *Do the age and the (decision) competence of the patients differ?* In most cases, but not all, hymenoplastic operations are performed on quite young females. Due to their low age, we might question the patients’ decision-making competence. However, healthcare personnel who encounter these women consider them competent to make decisions and, for instance, capable of rationally calculating the pros and cons of following the norms and deciding to have a hymen reconstruction or to abstain from it and abandon their families [3]. If not of age to consent, they may be able to assent to the procedure – exactly like young Jehovah’s Witnesses. However, it might be argued that these young females are too strongly influenced by the honour-related norms to be truly autonomous. However, it is hard to see why we should consider them more indoctrinated than young Jehovah’s Witnesses, who are, of course, also strongly influenced by their (religious) norms. These patients are usually considered competent and capable of rationally weighing up the pros and cons by either following the norms and deciding to have a bloodless operation or a blood transfusion and abandon their family [17]. When comparing persons in both groups who are considered old enough to make their own decisions in health care, there are no differences, although competency in general might be considered a relevant aspect.

Issue 7. *Offering alternative interventions*? It could be argued that we have an alternative treatment for young females who want a hymenoplastic operation, namely autonomy-empowering treatment, i.e., psychological treatment that enables the person in question to follow her own values and desires regardless of surrounding norms [18]. However, one could then ask why young Jehovah’s Witness patients should not be offered this as well. Moreover, autonomy-empowering treatment will not eliminate possible threats towards the females in question.

Issue 8. *Are there alternative ways to save the patient’s life*? The only way to save the Jehovah’s Witness patients (without putting them at serious risk of being ostracized from their communities) is to offer the additional treatments. However, as regards the young females from honour cultures, they can be encouraged to reject their honour-related norms and break loose from their community and, with the aid of the appropriate authorities, be shielded from the threat to their lives.

However, this is only an alleged difference. Jehovah’s Witness patients may also be encouraged to reject their religious norms and break loose from their community so as to enable them to accept blood transfusions.

Issue 9. *Do the sexes of the patients make a difference regarding fairness?* Obviously, honour-related norms are directed towards females, whereas the religious norms of Jehovah’s Witnesses concern both sexes. In practice, however, female Jehovah’s Witnesses are more often concerned, due to childbirth and the risk of bleedings. In principle, there is a difference here, but the question is whether or not it is relevant. If we assume that all fellow human beings are supposed to be treated equally within health care, there is no reason to show particular consideration for Jehovah’s Witnesses merely because the issue includes both sexes: for instance, sex, social position, and sexual inclinations are usually considered irrelevant when deciding priorities between treatments [19].

Issue 10. *Is there a difference in gender aspects of the norms?* The honour-related norms are primarily directed towards females and can be understood as an integral part of old patriarchal structures that discriminate against females by subjugating them to the males of the family. For instance, in a recently conducted survey, some physicians claimed that gynaecologists help to preserve and support these patriarchal structures by performing hymen restoration [20].

This argument is based on an empirical assumption regarding the consequences of gynaecologists granting young females’ requests for hymen restoration. However, we do not know if that is correct. On the contrary, we could also imagine the subsequent scenario: if all gynaecologists openly declared themselves willing to perform hymenoplastic operations on request, perhaps even patriarchs would understand that the norms (demanding virginity) are unfeasible in a modern society [20].

Nonetheless, one could still maintain that offering hymen reconstruction would *express* support for the patriarchal culture. This relates to a general bioethical discussion on so-called expressive arguments, that has been used for instance against prenatal screening for predetermined serious disabilities [21]. The heart of the argument is that by allowing certain (medical) practices, we are ‘sending out a message’, for instance that individuals with certain serious disabilities that health care choose to screen for are less worthy of protection. That is, by allowing and introducing a certain way of acting in health care, we show (whether or not we want to) that we accept certain norms of a problematic kind [21]. So, in this context, it could be claimed that by allowing and accepting hymen restoration, we express acceptance of the patriarchal norm that requires the young female to be able to demonstrate her virginity when being married.

Against the expressive argument, it must be noted that it is often unclear what kinds of beliefs and attitudes certain kinds of acts can be said to express. Providing hymen reconstruction does not necessarily express support for the patriarchal norms that recommend them. First, a certain act-type (like the act of performing hymen reconstruction) may be the result of a variety of attitudes and beliefs on part of the agent performing the act. Such an agent need not, of course, support (or intend to signal support) for patriarchal norms. As far as empirical findings suggest, the opposite seems true: many who perform these operations are simultaneously opposed to the patriarchal norms of honour-cultures [3, 20].

Second, a certain act-type (like the act of performing hymen reconstruction) may not necessarily be perceived as signalling certain beliefs or attitudes. One could actually maintain that society expresses repudiation of patriarchal norms by aiding young females in deceiving their representatives [20]. Also, one could say that by refusing hymenoplasty, society declares that it is the duty of young women, rather than society, to fight these norms, regardless of the cost to themselves. This seems questionable rather than laudable from a gender perspective.

The weaknesses of the expressive argument are especially noteworthy against the background of the overarching comparison in this paper, i.e., between aiding Jehovah’s Witnesses and victims of honour-related threats in health care. We have never encountered anyone claiming (and would be surprised if we ever did) that offering extraordinary measures for avoiding transfusions to Jehovah’s Witnesses would in any way imply expressing some sort of support for their religious beliefs and norms.

Actually, it seems both possible and defensible for physicians to perform hymen restorations and at the same time take other measures “to combat the outdated and discriminatory norms that are driving women to request such interventions in the first place” [22].

Issue 11. *Is there a difference between religious and cultural norms?* It might be held that the Jehovah’s Witnesses’ beliefs and norms are of a religious kind whereas the honour-related norms are not. Perhaps religious beliefs and norms are especially worthy of consideration.

However, we fail to see why one should think so. First, one has the problem of how to separate religious beliefs and norms from non-religious ones. Second, one has to put a case for the latter being more worthy of consideration than the former, which we (and others [23]) think is difficult, especially when they both regard basic beliefs and norms about how one should live one’s life and what norms one must never transgress. Third, honour-related norms too are sometimes a part of religious thought or tied to religious practice [1].

Issue 12. *Should society evaluate these subgroups differently?* It could be held that society should do more to protect Jehovah’s Witnesses than honour cultures, not because the former is an essentially religious subgroup and the latter not, but because there are reasons to think the latter is *less worthy of societal protection* or, simply, *worse*. For instance, (some) honour related norms are more clearly in contradiction to basic human rights by (sometimes) sanctioning killing, unlike the norms of Jehovah’s Witnesses.

First, this difference is at most a difference in degree: for instance, if one thinks that the right to abortion is a human right, some norms of Jehovah’s Witnesses are also in contradiction to human rights. Second, it is controversial whether a liberal society should rank different sub-cultures: one may argue that the liberal state should remain neutral with regard to the value-systems of different subgroups while at the same time, of course, incriminating all transgression of basic human rights [24]. In that case, society should not do more to protect the one group rather than the other, but still combat some of its behaviours.

However, even if one still thinks that honour norms are worse than Jehovah’s Witnesses’ norms (something to which we would actually agree) and even if one also would question this idea of the neutral state (an issue to which we remain neutral in this paper), there is the question of which *means* we should use to protect benign, so to speak, subgroups and combat malignant, so to speak, ones. It is far from self-evident that we should do so by refusing hymen restorations to females from honour cultures, which is the question at stake here. We think that most would agree that society should repudiate sub-cultural norms in violation of human rights and, at the same time, help the victims of such norms. For those who claim that offering hymen restoration would be incompatible with expressing repudiation of honour related norms, we disagree and refer back to the argument in Issue 10.

### Differences regarding our evaluation of the physicians

There are similarities and differences between the two interventions. But some of the differences are not morally relevant, and in most of the issues the interventions are, on closer inspection, similar. There is, however, at least one difference that might be considered relevant (patient safety) but it speaks in favour of hymen reconstruction. However, we do concede that this difference may not be considered important enough to justify treating the interventions differently. Consequently, if we accept the principle that equal cases should be treated equally, then health care staffs should offer these extraordinary treatments to neither of the groups or offer them to both.

If we accept this reasoning it might also be discussed whether it is acceptable to assess those who perform these practices differently. It is well-known that different specialties have different prestige; for instance the geriatric speciality is the lowest ranked one and brain surgery the highest ranked [25]. This is probably due to unofficial values within health care and society at large, for instance regarding older people, values that we, on closer reflection, would not want to carry any normative weight.

Such views are likely to also affect how we compare treating Jehovah’s Witness patients and patients from an honour-related culture. For one thing, we think it is safe to assume that performing a bloodless operation, which demands a skilled surgeon, is considered more prestigious than performing hymenoplastic operations. Moreover, the view of the different medical specialties differs: gynaecology is not as prestigious as surgery [25]. In addition, Jehovah’s Witnesses are Christians and used as paradigm cases of how we should treat norms and beliefs that we find hard to understand ourselves. We are used to them.

In comparison, the traditional honour-related norms are considered as belonging to an old-fashioned and distant shame culture, primarily associated with Islamic religion. Even though we might also find remnants of old honour cultures in Europe and Japan [26], the honour-related norms are not an accepted part of’western’ culture any more. Currently, the norms are primarily associated with violence against young females. For males to use violence against young female family members is not only considered wrong (rightly so, we believe), but incomprehensible and even disgusting [20].

These differences might also reflect on how we view the specialties: the surgeons who save a Jehovah’s Witness’s life are considered, if not heroic then at least praiseworthy; the gynaecologists are considered blameworthy. Is this reasonable and fair?

Jehovah’s Witnesses’ norms might also be considered as derived from outdated norms, irrational and dysfunctional. In fairness to the gynaecologists who perform hymen restorations, we should view and treat what they do in the same manner as we view and treat surgeons who perform bloodless operations on Jehovah’s Witnesses: if we castigate the gynaecologists who perform hymen reconstructions, we should also castigate surgeons who perform bloodless operations. And vice versa, if we praise surgeons who perform bloodless operations, we should also praise the gynaecologists who perform hymen operations.

## Summary

Since we did not identify any relevant differences between performing a hymen reconstruction due to honour-related norms and bloodless operations due to religious norms, we conclude that these patients should be treated equally. This means that neither group of patients should be offered operations or that both groups of patients should be offered operations. Similarly, there are no reasons for judging those who perform the operations differently. Both the gynaecologist and the surgeons should be blamed or praised (or neither) when saving patients’ lives.

## Authors’ information

NJ an associate professor in medical ethics at the Stockholm Centre for Healthcare Ethics (CHE) at Karolinska Institutet. His main research interests are in ethics and bioethics, genethics, and the intersection between political philosophy and medical ethics, e.g. autonomy and justice in health care.

NL is a professor of medical ethics and a GP. He is also the centre director of the Stockholm Centre for Healthcare Ethics (CHE) at Karolinska Institutet.
